# Comparison of the Success Rate of Endodontic Treatment and Implant Treatment

**DOI:** 10.5402/2011/640509

**Published:** 2011-06-15

**Authors:** Ranya Faraj Elemam, Iain Pretty

**Affiliations:** The University of Manchester, Oxford Road, Manchester M13 9PL, UK

## Abstract

Dentists and patients are facing a perplexity between saving a compromised tooth through endodontic treatment and restoration or by extraction and replacement with an implant. The purpose of this paper was to compare the success rates of these two treatments. Success was measured as the longevity of the tooth or implant. Studies which met strict inclusion criteria to ensure best evidence were included. Searches were performed in Ovid Medline, Pubmed, Scopus database, and the Cochrane Library. Evidence-based groups were formed following the assessment of inclusion/exclusion criteria. The overall success rates for primary endodontic, nonsurgical retreatment, and surgical treatment were (86.02%), (78.2%), and (63.4%), respectively, implants was 90.9%. In conclusion, choice between implant and endodontic therapy cannot be exclusively based on outcome as both treatments differ in the biological process, diagnostic modalities, failure patterns, and patients preferences. More research is required with improved study designs before long-term success rates can be compared.

## 1. Introduction

Preservation of a patient's natural dentition remains an important outcome in securing oral health. Endodontic treatments have been shown to successfully retain compromised teeth which were fractured, carious, or traumatised [1], for many decades. However, modern developments in implant provision provide greater choice for patients and clinicians, and, therefore, the decision between a root canal therapy and implant treatment is a commonly occurring dilemma in practice.

Outcomes of dental therapy as discussed in the literature fall into one of the four categories: success, survival with intervention, survival without intervention, and failure [2]. The majority of endodontic studies used the term “success” when describing treatment outcomes using clinical and radiographic parameters for the evaluation process. Recent studies pertaining to endodontic prognosis have adopted “strict” and “lenient” categories when illustrating successful outcomes. While complete radiographic healing and absence of any clinical signs or symptoms were characteristic of the “strict” category, reduction in size of radiolucency together with clinical normalcy defines the “lenient” criteria [3, 4]. In contrast, most outcome studies of implants used “survival” as the criteria of success. Thus, the difference in methodologies and definition of outcome measures makes comparison between the two treatment options very difficult for the researcher [5].

Investigators have disputed that survival/longevity is an improved measure of treatment success. Survival analyses permit the assessment of function over time and allows for the prediction of the longevity of a treatment modality [6]. Therefore, for purposes of this study, definition of success based on longevity/survival, that is, “tooth loss and time until repeat treatment for same or new condition” [7] was used.

This critical review compared success rates between root canal treatment and dental implant therapy based on the retention of a functional tooth or implant. The measurement of loss of an endodontically treated tooth and/or implant over time rather than measuring success rate based on strict or lenient criteria is more informative in deciding whether to endodontically treat or extract a tooth and replace it with implant.

The purpose of this literature review was to identify high-quality studies within the implant and endodontic literature that can provide information on the likely success as defined by survival rate of each treatment modality. The review will provide further information to clinicians and patients involved in the choice between endodontic therapy and implant replacement.

## 2. Methodology

Electronic searches were performed in Ovid Medline, Pubmed, Scopus database and the Cochrane Library; book searching as well as hand searching were undertaken using the following key words:

- Endodontics.mp. or endodontic
- Root Canal Therapy/or Tooth Apex/or Apicoectomy/ or apic (http://www.phru.nhs.uk/Pages/PHD/resources.htm) ectomy.mp. or Retrograde Obturation
- Dental Implants/or Dental Prosthesis, Implant-Supported/ or Dental Implantation/or Dental Implantation, End osseous/
- Retreatment/or endodontic retreatment.mp.
- Time Factors/or longterm.mp.
- Longevity.mp. or Longevity/
- Longitudinal Studies (Tooth or teeth) and (retain or retention).

### 2.1. Study Selection and Data Analysis

A search strategy was developed for 3 disciplines: endodontic treatment (primary endodontic treatment, nonsurgical retreatment, and surgical treatment), implant treatment, and implant versus endodontic treatment. Articles were first selected according to titles and abstracts, and then they were fully reviewed to ensure that they met the inclusion/exclusion criteria. Subsequently, evidence based groups were constructed according to the treatment options with the following information that were categorised: author's name, year of publication, study type such as (prospective/retrospective, cohort, clinical trial) number of units (teeth/implants), success rate, study setting, practitioners, and length of followup. Excluded studies with reasons for their exclusion were also grouped. Articles were then appraised using CASP checklists and overall comments about each studies quality were noted. Subsequently, the estimation of mean and standard deviation of both success rates and following-up period of time were calculated for each group, and a summary table created to provide the overall success rate of each treatment modality.

Inclusion criteria included studies with prospective or retrospective data, using different materials and/or techniques. Included papers mentioned success rates in percentages. Searches were limited to papers written in English with human subjects and published between 1998 and 2008. Studies were suitable for inclusion if they were reported with a minimum 4-year followup to ensure that they met the definition of success.

The exclusion criteria consisted of studies that failed to meet the inclusion criteria. If a paper did not define criteria for success/survival outcomes nor did it report follow-up period of time, it was excluded. Likewise, if the followup was less than 4 years, the paper was also discounted. The papers that neither stated the percentage of success rate nor used human subjects were similarly discarded. All systematic reviews, literature reviews, case series, study reports and opinion only articles were assessed but not included within the analysis. Papers that dealt with the identification of bacterial species were also excluded.

## 3. Results

Following the initial search 108 studies were identified. (50) articles were excluded after the initial screening, and (58) were retained where they were divided into (36) endodontic related papers, (13) papers were associated with implant, and (9) were dealt with the comparison between the two treatments (group 1).

Following the full text review of the (36) endodontic articles and (13) implant studies, (26) papers were rejected. They were grouped with the reasons for rejection. The remainder of the (23) publications were considered relevant based on the inclusion/exclusion criteria. Those papers were split into (5) conventional endodontic treatment, (6) nonsurgical retreatment, (4) surgical endodontic treatment, and (8) implant treatment papers which were presented in groups 2, 3, 4, and 5; these data are summarised in Table 1.

### 3.1. Exclusion Papers

Four groups were constructed according to the treatment type. 

 The primary endodontic treatment exclusion group enclosed three papers, the reasons of the exclusion were the short period of the observation time [9] and the literature review design [4, 10].

The second time root canal treatment exclusion group contained eight papers of which exclusion was because of the study designs case report and literature reviews [11–15]. The period of followup was less than 4 years [16], a further paper was excluded as the author didn't clearly separate the initial and secondary treatments samples [17]. The surgical endodontic treatment group included 9 articles. Papers were excluded due to: a followup period of time was less than 4 years, [18, 19], success rate was not described [20, 21], paper did not state the success rate at the exact time of 4 years [22], papers limited to case series [23, 24] literature or systematic reviews [25, 26]. Six implant papers were excluded two of which did not state success rate [27, 28], success rate not at four years [29] and review studies [30–32].

### 3.2. Endodontic versus Implant—Group 1

Six articles included were chart reviews, they were considered as-low quality papers based on CASP.

In two of them, the treatment procedures took place in universities [2, 8], while one mentioned speciality clinics [5]. Success rates were cited as 98%, 73.5% for implant, 99.3%, 82% for endodontic [2, 5].

### 3.3. Primary Endodontic Treatments—Group 2

Five papers were included. All samples were a combination of tooth type (single-rooted and multiple-rooted teeth). The highest success rate was 97% [33], and the lowest success rate was 73% [34].

Most of the treatment procedures were performed in teaching hospitals by graduate and postgraduate students [3, 34, 35] only two studies reported activities within specialist primary care settings [33, 36]. According to CASP, most of the papers were of a high quality [3, 33, 34]. However, the others were of a moderate quality [35, 36].

### 3.4. Secondary Endodontic Treatments—Group 3

This group contained 6 papers. All dealt with multirooted teeth except one paper which had a single-rooted sample [37]. The sample sizes varied between 86 to 624 teeth [36, 38].

The success rate reported varied, the highest rate of 95% was reported by Fristad et al [39] while the lowest rate was 59.5% described by Kvist and Reit [37]. Three papers reported endodontists as a treatment provider [36–38], whereas the remainder described nonspecialists, who were graduate, postgraduate, and general dental practitioners [39–41]. Most of the treatment procedures were undertaken in teaching hospitals while one was set in primary care [36]. Moderate quality papers were dominant in this collection (4 out of 6), with one high-quality paper and one low quality based on the CASP assessment. Three prospective cohort designs were identified [38–40], one randomized control trial [37], and the remaining were retrospective study designs [36, 41].

### 3.5. Endodontic Surgery Treatments—Group 4

Four papers were included. All 1005 teeth were multirooted. The success rate ranged from 27.84% [42] to 80% [43]. With the exception of one paper reporting that the treatment was conducted by undergraduate students [44], most of the treatment was performed by specialists. The treatment procedures were carried out in teaching hospitals in two papers [42, 44]. in primary care in one paper [43], and the setting was not mentioned in the final paper [45]. There were two low-quality papers among this group [42, 43], one paper was of a high quality [45] while the other was moderate in quality [44].

### 3.6. Implant Treatment—Group 5

8 papers were included in this group comprising of a total 1047 implants. Two studies had utilised bone grafting treatment before the insertion of implants [46, 47]. The success rate ranged from 74.6% to 99% [46, 48]. In 6 papers, the treatments were carried out in universities and only two of them mentioned that the providers were specialists [48, 49]. Two studies reported setting as primary care [49, 50]. According to CASP, the majority of the papers were indicated to be of high quality [46–48, 50, 51], only one was moderate [49] and two were low [52, 53].

The results of these assessments.

## 4. Discussion

The articles reviewed within this work varied considerably in their findings regarding the long-term success of endodontically treated teeth and implant treatment (Table 1). This wide variation might be related to differences in the size of samples, type of the teeth, indications for the operation and the treatment procedures, the specialist status of the operator, and the materials used.

The influence of different pretreatment and treatment variables were also diverse. These variables were preoperative periapical status preoperative pulp vitality, tooth type, quality and extension of instrumentation, type of coronal restoration used, and type of inter-appointment medicament. While a number of studies [33–36, 38–41, 45] found that some of these factors did have a statistically significant impact on the success of the root canal treatment, others found that the same variables had no significance [42–44]. Different factors have been revealed to contribute to the predictability of implants such as implant site, systemic diseases, smoking, and bone quality [2, 5].

Most of the papers were retrospective studies, which are limited by the lack of controls and randomisation as well as other patient selection factors. Such studies are prone to bias especially that associated with treatment, recall and outcome [54].

For primary endodontic therapy there are several reported factors that have been shown to influence success rate. The presence of periapical radiolucency affected the success rate as reported by [35, 36], the latter study concluded that a higher success rate was observed in teeth without periapical radiolucency as compared to those with periapical radiolucency. Previous papers had regarded the presence of periapical radiolucency as an indication for retreatment [55, 56]; therefore, they considered the periapical radiolucency as a failure of primary endodontic treatment. Dammaschke [35] suggested that an evaluation period of 1-2 years was sufficient to observe the success rate in teeth without periapical periodontitis, while in the presence of periapical lesions, a period of 2–5 years may be needed [37].

Coronal coverage was found to significantly influence the outcome of endodontic treatment [33, 36]. When teeth were restored with coronal coverage, a higher success rate was shown as compared to those without coronal coverage [36]; the extraction of the endodontically treated teeth without coronal restorations was 6.2-fold greater than in those with crowns [33].

It was reported that the treatment provided by specialists and students yielded high success rates [3, 33, 35], and in the case where students showed a higher success, it was explained by the fact that the students were more careful during the treatment of more complex molars [35]. However, previous papers stated that specialists can achieve higher success rates than those achieved by student [57].

Multirooted demonstrated lower success rates than single-rooted teeth [3] this difference may be due to the anatomic difficulties in molars (curved roots) and limited access to the posterior area [35].

Higher healing rates were observed in teeth treated in two or more sessions than those treated in a single session, but this was not statistically significant [3]. This finding differed from Imura's study [36] which explained the effect of the number of visits as being probably related to the intracanal medicament used to dress the canals.

Failure of the initial endodontic treatment was considered in the following cases: extraction of the tooth because of pathology of endodontic origin, surgical or nonsurgical retreatment, and existing periapical radiolucency of any size. Failure can occur early or late within the treatment cycle. Early failures were usually due to improper initial treatment [34], whereas several factors could contribute to late failures, most importantly reintroduction of microorganisms in the root canal system due to lack of coronal coverage [34]. Age was not statistically significant factor, though failure rate was high among the elderly population [35]. This higher failure rate was explained as being due to the widespread of periodontal diseases that cause bone loss and subsequent extraction of the tooth in older people.

In nonsurgical retreatment, the success was generally regarded to be lower than that of primary treatment [40]. Low success rate was particularly noticeable in the presence of apical periodontitis [40, 58]. It was observed that in the teeth with apical periodontitis where the previous root filling was insufficient, the outcome of retreatment was better than that of the teeth with sufficient filling. In the cases where fillings were insufficient, the canals were the source of infection, when retreated they could be adequately disinfected and obturated resulting in a favourable healing. When the previous root filling was sufficient, the cause of persistent radiolucency could be extraradicular infection [59], a true cyst [60], or presence of foreign body reaction [61]. These above mentioned situations would not respond to orthograde retreatment [40]

Length of root canal filling also was found to influence the outcome of the endodontic treatment [41]. The most successful outcomes were associated with teeth without apical extrusion of the filling material [39]. The overextension of filling materials was combined with delayed healing [61, 62]. This delay required a longer observation period.

There was a statistically significant difference in the survival time between asymptomatic and symptomatic teeth, with the asymptomatic teeth having increased survival times [41], this was consistent with that reported by Friedman [63]. In contrast, previous studies reported that preoperative clinical symptoms had no influence over the outcome of endodontic treatment [64–66].

Perforation was reported to negatively affect the prognosis of the retreatment. Large size, more coronal, and older perforations showed poorer prognosis [40]. Vital teeth showed better survival than nonvital. [41]. However, other studies showed no difference [64, 67]. Molar teeth showed significantly lower success rates than premolars and anterior teeth in nonsurgical retreatment [36]. Additionally, it should be noted that there was no significant difference in the success rate of the treatment which was carried out by students and those by qualified dentists.

In a study comparing the primary and secondary endodontic therapy, it was shown that the success rate of primary endodontic treatment was 94.0%, while that of nonsurgical retreatment was 87.9% [36]. The lower success rate of secondary treatment in this study may be due to the incomplete elimination of certain microorganisms are known to be common in such cases, for example, E. faecalis, the elimination of this microbe could be difficult because of its resistance to some disinfectants used during the treatment, particularly calcium hydroxide [68, 69]. It has been postulated that E. faecalis may be able to invade the dentinal tubules and adhere to collagen in the presence of human serum [70].

Endodontic surgery has been indicated to conserve teeth with persistent periapical lesions or following a failed root canal retreatment [71]. Its outcome had been measured with a reported success rate ranging from 27.84% to 80% [42, 43].

The least favourable results were founds when teeth underwent a resurgery whereas the best survival rate tended to be found in those teeth where the root filling and surgery were carried out at the same time [44, 72]. Complete canal debridement was an important part for success in both nonsurgical and surgical root canal treatment [73]. The success of periapical surgery was improved with smaller periapical lesions and smaller apical resection and did not depend on the magnitude of retrograde filling [45].

The influence of different periapical surgical techniques and different retrofilling materials used for managing the root canal system were neglected in most of the articles; however, the standard and the ultrasonic methods have been compared in one study which showed that 85% of success rate was achieved in the cases that were treated by the ultrasonic technique and 68% was the success rate in the cases treated by rotary instruments [43]. This difference was explained as the ultrasonic treated cases were filled with EBA which has a better success rate than amalgam which used in the other group. The success associated with the ultrasonic might be related to the less bevelled root resection which may create less apical dentin permeability [74]. 

Factors like root anatomy, proximity to vital structures, and clinical accessibility will increase the level of technical difficulty of periapical surgery of molars [44]. Therefore, tooth type and position have a significant impact on the success rate of endodontic surgery [43]. In this study, the success rate for maxillary teeth was (78%) and that for mandibular teeth was (66%).

As far as the skill of the operator was concerned, one study suggested that students outperformed senior staff surgeons [44]. This was explained by that difficult cases which were judged to have a lower chance of success rate were performed by a staff member. Both endodontists and oral surgeons mentioned in the rest of the articles as the operators, although their approaches were considerably influenced by different philosophies, culture, training pathways, and attitudes [75], they achieved similar results [42].

Failure in the endodontic surgery was reported in one study as two distinct types: emergent and inconspicuous [44]. Emergent failures occurred immediately after the treatment and the teeth were either reoperated upon or extracted. Additionally, this type of failure could be recognized by symptoms or emergencies leading to either extraction or decision to intervene. It was debatable that if these cases had been reviewed before symptoms occurred, failure might have been diagnosed earlier. Success declined with the presence of intraradicular posts, when the coronal restoration had a poor marginal seal and presence of an inadequate apical seal [42]. Low survival time was also described in teeth with noticeable marginal bone loss [44]. In this study, first-time surgical endodontic treatment survived significantly longer than resurgery cases.

Implant survival was reported to be influenced mainly by the quality of bone [46]; in some cases where bone level was insufficient to insert implant, bone grafting was required which resulted in higher success rates [46, 47]. Furthermore, survival rate was significantly affected by the presence of peri-implantitis, which is defined as “an inflammatory process affecting the tissues around an osseointegrated implant in function, resulting in loss of supporting bone” [76].

Failure was diagnosed by bone loss, incident of deep pocketing, and the amount of bleeding on probing [77]. In some patients, recurrent inflammation and bone loss were reduced with early diagnosis and treatment to isolate and decrease the damage. Accordingly, regular examinations can influence longevity of an implant [52].

10-year success rate of the implants inserted in the periodontally compromised patients was lower than the 10-year success rate that has been reported for implants inserted in healthier subjects [50]. That was in contrast to [49, 53] where implants were successfully inserted in periodontally susceptible patients with 49.7–95% success rate. It could be explained here that the mean peri-implant bone loss was very narrow statistically, and for this reason, the implant was considered as highly predictable. Yet, this fact did not prevent the possibility of occurrence of peri-implantitis or alveolar bone loss around single implants in susceptible patients. In such patients, it may, therefore, be important to evaluate the patient's risk for peri-implantitis and carry out a regular periodic recall as a part of an implant program [52]. Nonetheless, one study showed that the presence of a well-functioning marginal mucoperiosteal osseous barrier zone at the implant mucosa interface considered as a vital requirement for long-term favourable outcome of implant treatment [47].

Significant factors affecting bone loss around implants (smoking, health problems, and attachment level) were identified [50, 53]. In a study which had a 5-year survival rate of 99%, all patients treated with dental implants were healthy and nonsmokers [48]. Age, gender, or implant type did not have any significant influence on the survival of the implants [50].

Two papers mentioned the influence of implant location on the success rate, one of these two papers concluded that implants placed in the maxilla presented with deeper periodontal pockets and greater attachment loss over time than those placed in the mandible [53]. The other study where the implants successfully placed in reconstructed maxilla and mandible using free bone graft, the lost implants in each case were usually located near the middle area of the reconstructed part [47].

Implant failures were generally caused by the inability of the body to tolerate the implant material and hence failure of osseointegration [5]. Failures can occur during the treatment stage or later during the maintenance phase. Early failures were usually the result of insufficient osseointegration and were attributed to the formation of a fibrous connective tissue interface between the implant and recipient bone surface [78]. Failures during the maintenance phase were in general caused by bacterial infection or biomechanical factors that gradually affected the implants osseointegration in a route similar to that of periodontal disease around the implant [79].

## 5. Summary

A high success rate of 86%, 78.2% in primary and secondary endodontically treated teeth described in this paper supports the benefit of endodontic treatment and suggests that the patients should be offered this treatment before tooth extraction. Nonsurgical retreatment should be regarded as the first choice when primary treatment fails [42] especially in cases of poor root canal restorations and where renegotiation of the root canals is possible [80]. Surgical treatment in such cases will probably result in the development of a new or recurrent infection [38]. Furthermore, it was reported that when nonsurgical root canal treatment was performed in teeth that had been previously managed with apical surgery, the success rate was lower than in those previously endodontically treated [38]. However, periapical surgery remains an alternative option for the management of cases in which nonsurgical retreatment was not possible [42]. A remarkable finding was that the sizes of the sample were relatively small in all surgical papers compared to secondary treatment papers. This may be related to the increasing use of orthograde retreatment, where it was indicated in most cases of endodontic failures [81].

## 6. Conclusion

When teeth can be treated successfully with endodontic therapy, primary endodontic treatment would normally be the first choice treatment, should this fail, nonsurgical retreatment should be considered. Surgical treatment can often be performed subsequently, and it should attempted before losing the teeth by extraction. Failure should always be considered in any treatment plan and the options to deal with it discussed. Implant treatment can often be a suitable alternative and should be considered and offered to all patients when appropriate.

The current work suggests that both main treatment modalities (endodontics and implants) have value. The quality of the evidence is good to moderate, although that surrounding the use of implants is weaker. In order to provide properly informed treatment decisions and ensure that patients are fully consented and aware of their treatment options, further empirical evidence is required.

## Figures and Tables

**Table 1:** 

Treatment	Number of studies (inclusion and exclusion)	Number of teeth/implant	Average followup	SD of mean followup	Mean survival	SD of mean survival
Primary endodontic treatment	5/3	1,465,158	6.7	2.8	86.02%	9.7
Secondary endodontic treatment	6/8	1561	8.7	7.5	78.2%	14.7
Surgical endodontic treatment	4/10	1005	7.5	3	63.4%	23.9
Implant treatment	8/5	1047	6.8	2.5	90.9%	7.6
